# Dynamics and immunomodulation of cognitive deficits and behavioral changes in non-severe experimental malaria

**DOI:** 10.3389/fimmu.2022.1021211

**Published:** 2022-11-24

**Authors:** Pamela Rosa-Gonçalves, Luciana Pereira de Sousa, Aline Barbosa Maia, Flávia Lima Ribeiro-Gomes, Caroline Cristhiani Tavares de Lima Gress, Guilherme Loureiro Werneck, Diogo Onofre Souza, Roberto Farina Almeida, Cláudio Tadeu Daniel-Ribeiro

**Affiliations:** ^1^ Laboratório de Pesquisa em Malária, Instituto Oswaldo Cruz, Fundação Oswaldo Cruz (Fiocruz), Rio de Janeiro, Brazil; ^2^ Centro de Pesquisa, Diagnóstico e Treinamento em Malária, Fiocruz and Secretaria de Vigilância em Saúde, Ministério da Saúde, Rio de Janeiro, Brazil; ^3^ Departamento de Epidemiologia do Instituto de Medicina Social da Universidade do Estado do Rio de Janeiro and Instituto de Estudos de Saúde Coletiva da Universidade Federal do Rio de Janeiro, Rio de Janeiro, Brazil; ^4^ Departamento de Bioquímica, Instituto de Ciências Básicas da Saúde, Universidade Federal do Rio Grande do Sul, Porto Alegre, Brazil; ^5^ Departamento de Ciências Biológicas, Programa de Pós-Graduação em Ciências Biológicas, Núcleo de Pesquisas em Ciências Biológicas, Universidade Federal de Ouro Preto, Ouro Preto, Brazil

**Keywords:** behavior, cognitive dysfunction, diphtheria-tetanus vaccine, immunomodulation, non-severe experimental malaria

## Abstract

Data recently reported by our group indicate that stimulation with a pool of immunogens capable of eliciting type 2 immune responses can restore the cognitive and behavioral dysfunctions recorded after a single episode of non-severe rodent malaria caused by *Plasmodium berghei* ANKA. Here we explored the hypothesis that isolated immunization with one of the type 2 immune response-inducing immunogens, the human diphtheria-tetanus (dT) vaccine, may revert damages associated with malaria. To investigate this possibility, we studied the dynamics of cognitive deficits and anxiety-like phenotype following non-severe experimental malaria and evaluated the effects of immunization with both dT and of a pool of type 2 immune stimuli in reversing these impairments. Locomotor activity and long-term memory deficits were assessed through the open field test (OFT) and novel object recognition task (NORT), while the anxiety-like phenotype was assessed by OFT and light/dark task (LDT). Our results indicate that poor performance in cognitive-behavioral tests can be detected as early as the 12^th^ day after the end of antimalarial treatment with chloroquine and may persist for up to 155 days post infection. The single immunization strategy with the human dT vaccine showed promise in reversal of long-term memory deficits in NORT, and anxiety-like behavior in OFT and LDT.

## Introduction

Malaria, an infectious parasitic disease caused by protozoa of the genus *Plasmodium*, is a major public health issue (1). The clinical course may present in the classic form of fever, chills, sweats and headache or evolve to complicated and severe presentations of the disease, such as cerebral malaria (CM), a lethal form of *P. falciparum* infection (2). Cognitive deficits and behavioral changes are associated with CM and have also been recorded after episodes of non-severe malaria (nSM), the most prevalent clinical form of malaria, as well as in murine experimental models (3–13). However, so far there is no effective treatment for these sequelae, the mechanisms and consequences of which require further study.

The immune and nervous systems are plastic cognitive systems that communicate and interrelate, both being capable of modifying cognitive function. Immune stimuli are known to modulate neurogenesis, synaptic plasticity, and cognitive and behavioral functions (14–17). We have previously shown that active immunization by different integrated stimuli (anti-inflammatory inducers of the Th2 response profile) initiated early after treatment of a nSM episode caused by *P. berghei* ANKA (*Pb*A) in C57BL/6 female mice, can abrogate the late expression of deficits in cognitive function and can also inhibit the manifestation of an anxiety-like phenotype (18). The present study: i) analyzes the dynamics of cognitive deficits and anxiety-like phenotype evolution following *Pb*A experimental nSM, and ii) investigates the effect of isolated immunization with the adult human diphtheria-tetanus (dT) vaccine on these parameters.

## Materials and methods

### Animals and parasite

All animal procedures were carried out in accordance with the ethical principles and animal welfare practices approved by the ethics committee of the *Instituto Oswaldo Cruz* (*CEUA-IOC, Fiocruz*, license numbers L-010/2015, L-004/2020). Seven- to eight-week-old female C57BL/6 mice (n = 209), weighing 18-20 g, were provided by the *Instituto de Ciência e Tecnologia em Biomodelos* of the *Fundação Oswaldo Cruz* (*ICTB-Fiocruz*). Mice were maintained in controlled conditions of humidity (50% ± 10%), temperature (20° C ± 2° C) and light (12-hour light/dark cycle), with access to chow and water *ad libitum* in polypropylene cages with one Igloo™, with four to five mice per cage, kept in racks with an air filtration system in the animal facility of the *Centro de Experimentação Animal* of the IOC (*CEA, IOC-Fiocruz*), located at *Pavilhão Leônidas Deane*. One mouse was excluded due to compromised welfare. Parasite infection was performed with the ANKA strain of *Plasmodium berghei* (*Pb*A), transfected with green fluorescent protein (GFP) from the Malaria Research and Reference Reagent Resource Center (MR4)-BEI Resources.

### Infection and treatment

Mice were inoculated intraperitoneally (ip) with 10^6^ *Pb*A-GFP-infected red blood cells in 100 μL of phosphate buffered saline (PBS). This parasite/host combination corresponds to the classic model for the study of CM (19, 20). The concentrations of parasitized red blood cells (pRBC) for the inoculation were obtained from the blood of eight “passage” mice previously infected with 150 μL of an aliquot of the strain thawed from liquid nitrogen, kept in a cryopreservative solution (0.9% sodium chloride, 4.2% sorbitol, 20% glycerol). Five days was enough to reach percentages of parasitemia above 5% and the whole blood was collected by cardiac puncture under ketamine and xylazine anesthesia for infection (infected groups), after adjusting the concentration of the intended inoculum to 100 μL. Parasitemia measurements were obtained by flow cytometry or microscopy of blood on slides fixed in methanol, stained with Giemsa’s solution and observed under an optical light microscope (Olympus BH-2) to quantify the percentage of infected erythrocytes (1,000 erythrocytes were counted per blood smear).

Non-infected or non-infected/non-immunized control or non-infected/immunized mice were injected with PBS only using the same procedures. After infection of mice, measurement of parasitemia in the experimental group was performed to verify the effectiveness of the infection. Blood drops were collected by minimal section of mouse tails for blood distension analysis, or were diluted in PBS for later acquisition in a Cytoflex S flow cytometer for obtaining the percentage of GFP^+^ red blood cells. Control mice were submitted to the same procedure of minimal tail sectioning. The clinical manifestation of CM can occur from the fifth day after untreated infection and is more evident from the sixth day on, when, gradually, neuropathological signs (ataxia, convulsion, paralysis, coma and death) appear (21).

In this study, infected mice showed increased levels of parasitemia from the third to the fourth day after infection with parasitemia around 2.5%, when treatment with chloroquine (CQ, Farmanguinhos-Fiocruz) was started, before any clinical signs of severe malaria were apparent, as described by De Sousa et al. (6). CQ treatment was administered (in 200μL of 25 mg/kg solution in PBS) once a day by oral gavage, for seven days. At the end of treatment, blood was screened by microscopy for the presence of parasites to confirm the effectiveness of the treatment in reducing parasitemia. Three mice were excluded due to recrudescence of parasites following CQ treatment. Although CQ treatment does not influence the behavioral performance of mice in tests performed approximately 77 days after treatment (6), we treated groups of non-infected mice with PBS or with CQ to screen for the presence of any CQ effect on cognitive and behavioral parameters 12 days after treatment.

### Immune stimuli

Two weeks after the end of treatment, two distinct immunization strategies capable of inducing type 2 immune responses were adopted: a) vaccines and immunogens [referred to as T2 strategy, firstly described by De Sousa et al. (18)] and b) adult human diphtheria-tetanus (dT) vaccine. The integrated stimuli “a” encompassed the dT vaccine, the same used in the *Programa Nacional de Imunizações* (*PNI* – *Ministério da Saúde*, Brazil). It is an inactivated vaccine, manufactured by Biological E. Limited (lot: 221500317C) and was administered to mice in a three-dose regimen *via* the dorsal subcutaneous route, with an interval of 21 days between each dose of 100 μL containing 0.4 Lf of diphtheria toxoid and 1.76 Lf tetanus toxoid, adsorbed on aluminum phosphate (AlPO4 ≥ 0.3 mg), with thiomersal preservative (0.01%) kept at 2 to 8°C until the time of administration. In addition to the dT vaccine, the T2 strategy also comprises three doses of the c-terminal portion of *P. falciparum* Merozoite Surface Protein 3 (*Pf*MSP-3), administered subcutaneously at the base of the tail, with an interval of 21 days apart. Each 100 μL dose contains 10 μg of the immunogen diluted in PBS and adsorbed on 70% Montanide™ ISA 720VG ST adjuvant manufactured by SEPPIC S.A. (lot: 2587851). The T2 strategy also included white chicken egg ovalbumin (OVA, Sigma-Aldrich A5503-50g), administered in three doses of 100 μL, one by the dorsal subcutaneous route and two by the ip route, with an interval of seven days between doses and starting 24 hours after the last dose of *Pf*MSP-3. Each dose contains 50 μg OVA dissolved in PBS and adsorbed on 5 mg/mL aluminum hydroxide [Al(OH)_3_]. The isolated dT vaccine strategy was performed as described in “a”.

### Experimental protocol for the study of the dynamics and immunomodulation of behavior in malaria

**Figure 1:**
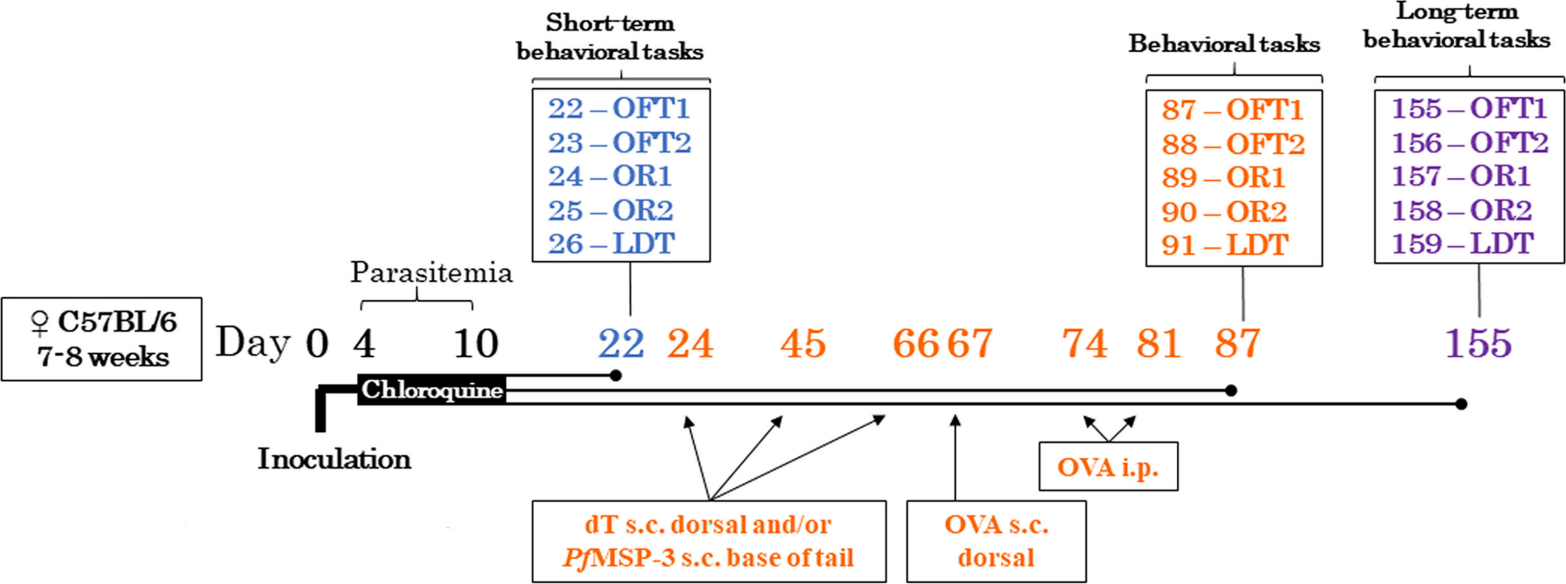
Experimental design. C57BL/6 mice were infected with 10^6^ parasitized red blood cells with *Plasmodium berghei* ANKA on day 0 or injected with the vehicle (PBS control) intraperitoneally. Four days post infection, both mice were treated with chloroquine 25mg/kg *via* gavage for seven days and parasitemia was measured at the beginning and end of treatment, and after behavioral assays. To assess the evolution of cognitive-behavioral performance, cohorts of control (CTRL) and infected (INF) mice were evaluated 12 (cohort 1) and 145 (cohorts 2 and 3) days after the end of treatment in behavioral tests. To evaluate the immunomodulatory effect, control and infected mice were immunized or not with the dT vaccine immunization strategy. For comparative purposes, a group of infected mice was immunized with the type 2 immune response profile immunization strategy. All mice were evaluated in behavioral tests; PfMSP-3 – *Plasmodium falciparum* Merozoite Surface Protein 3, dT vaccine – diphtheria-tetanus vaccine, OVA – white chicken egg ovalbumin, s.c. – subcutaneous, i.p. – intraperitoneal; OFT – open field test, OR1 – object recognition 1 (training session), OR2 – object recognition 2 (test session), LDT – light/dark task.

Groups of C57BL/6 mice were randomly divided into the control (CTRL) and infected (INF) groups and inoculated *via* ip with 10^6^ pRBC by *P. berghei* ANKA-GFP (INF) or injected with PBS (CTRL) (**Figure 1**). On the fourth day post inoculation (dpi), both groups of mice received antimalarial treatment with CQ 25 mg/kg, *via* gavage, once a day, for seven days. Parasitemia was measured on the first (D4) and last day (D10) of treatment, and after behavioral assays. To assess the dynamics of cognitive and behavioral performances, CTRL and INF mice groups were randomly divided into three cohorts of 40 [12 days post-treatment (dpt), cohort 1], 24 (145 dpt, cohort 2) and 35 (145 dpt, cohort 3) animals. They were subsequently submitted to a sequence of behavioral tests: open field test (OFT), novel object recognition task (NORT) and light/dark task (LDT), at 22-26 dpi, (12-16 days post-treatment, endpoint referred to as “12 dpt”) or at 155-159 dpi (145-149 dpt, endpoint referred to as “145 dpt”). Three mice had low levels of parasitemia and were excluded from the analyzes. To assess the effect of a single immunogen in reversing malaria parasite infection related pathology, mice were subdivided in five groups: control (CTRL, n=19), control immunized with the dT vaccine (dT, n=19), infected (INF, n=24), infected immunized with the dT vaccine (INF- dT, n=19) and infected immunized with T2 stimuli (INF-T2, n=19) groups and submitted to the immunization protocol 14 days after the end of the antimalarial treatment. These groups were submitted to the behavioral tests OFT, NORT and LDT 87 dpi (77 dpt). All CTRL groups were age-matched, mock-infected, mock-immune stimulated and treated with CQ whenever applicable, and treated in the same manner as the experimental groups.

### Cognitive and behavioral analysis protocols

**Figure 2:**
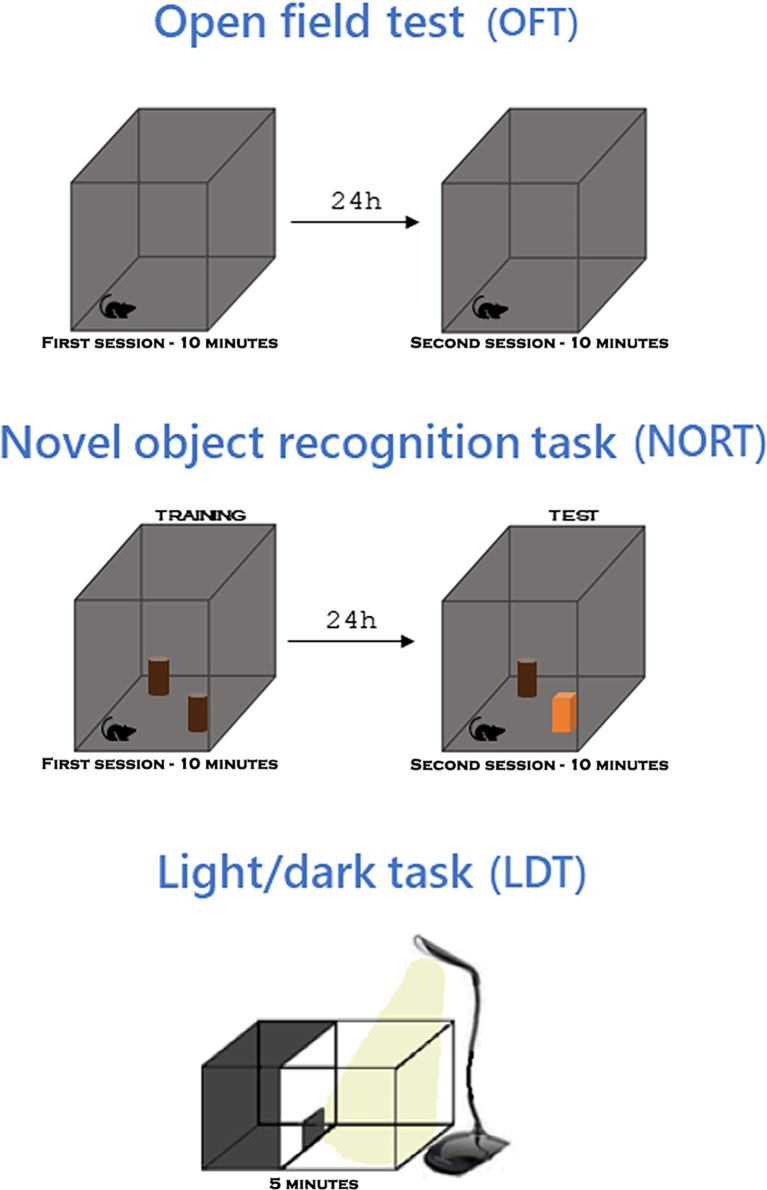
Behavioral tests. In the open field test (OFT), the animal explores the apparatus for 10 minutes and 24 hours later explores again. In the novel object recognition task (NORT), performed in the same apparatus as the OFT, the animal explores two identical objects for 10 minutes and 24h later it explores them again, one of the objects being replaced by a different one. In the light/dark task (LDT), the animal explores an apparatus with two compartments, one dark and one light, for 5 minutes. For more details read section Materials and methods on Cognitive and behavioral analysis protocols.

The sequence of behavioral tests involved evaluation of parameters related to locomotor activity, cognitive performance, and anxiety-like behavior, as reported by De Sousa et al. (6, 18) (**Figure 2**). The sequence involves an increasing order of aversiveness, to minimize the impact of one task on the next. Behavioral tests were carried out in the afternoon in an environment with 60 lux light intensity provided by incandescent lamps, and mice were acclimatized for two hours prior to testing with access to chow and water *ad libitum*. Mouse performance was recorded by a video camera positioned above the device, connected to a laptop computer. Automated acquisitions and analyzes were performed using AnyMaze^®^ version 5.1 software (Stoelting Co., Wood Dale, IL), while manual analyzes were performed by a researcher blinded to group assignment. The devices were sanitized with 70% alcohol and dried, before and after use by each mouse.

### Open field test

The OFT was adapted from De Almeida et al. (22), as described by De Sousa et al. (6, 18). Mice in cohorts 12 and 145 dpt were individually placed in the apparatus close to the side wall. The OFT arena consist of a gray acrylic box (50 × 50 × 50 cm). Each mouse was free to explore the apparatus for 10 minutes in the first OFT session (OFT1). After 24 hours, they were submitted to a second OFT session (OFT2), for 10 minutes. The parameters determined by two consecutive OFT tasks were: i) locomotor activity measured by total distance traveled in both sessions, ii) long-term habituation memory to a novel environment [reflected by decreases in the total distance traveled in OFT2 compared to the OFT1, as previously characterized by De Almeida et al. (22), since the environment, already explored in OFT1, does not correspond to a novelty and is consequently less explored in OFT2], and iii) the anxiety-like phenotype by time and distance traveled in the center zone of the OFT1 apparatus. Cohort 3 of 145 dpt was analyzed in a smaller acrylic box (30 × 30 × 30 cm). All the data from above mentioned parameters were obtained by the AnyMaze^®^ 5.2 software.

### Novel object recognition task

The NORT was adapted from De Almeida et al. (23), as described by De Sousa et al. (6, 18). In this task, the recognition memory is evaluated based on the mice performance due to the animal’s natural preference for novelty. NORT was performed 24 hours after the OFT2 session and was carried out in the same OFT arena, in order to reduce spontaneous anxiety levels associated in novel environments (6, 18). Mice were always placed individually, facing the apparatus wall and equidistant from the two objects, and could freely explore for 10 minutes. The training session consisted of placing two identical objects (FO1 and FO2). After 24 hours (to assess long-term memory), mice were again individually placed in the arena, with one of the familiar objects being exchanged for a different one (in terms of texture, shape and size) – named novel object (NO). Exploration was defined as the time a mouse spent with its snout facing the object at a distance ≤ 2 cm and/or touching the object with the snout or forepaws. The exploration time in each object and its percentage were calculated by dividing the exploration time of the novel or familiar object by the sum of the exploration time for the two objects, multiplied by 100. The exploration time was manually measured by a blinded to treatment researcher, using a digital stopwatch, by observing video recordings of the sessions. Cohort 3 NORT performance was analyzed in a small acrylic box (30 × 30 × 30 cm). Six mice from cohort 1, 11 from cohort 2 and 12 from groups of immunization protocol were unable to explore the objects and were excluded from the analysis according to Lourenco et al., with minor modifications (24). Based on mice ethology, it is expected that the exploration period dedicated to the novel object will be longer than that of the familiar object.

### Light/dark task

The LDT was adapted from De Almeida et al. (25) as described by De Sousa et al. (6, 18). This test is based on the natural preference of mice for closed areas over open and lit (exposed) ones (26). This test was carried out in an acrylic box (45 cm long × 27 cm high × 27 cm wide) divided into light and dark compartments (the latter being smaller), with the presence of a portal that allows the transition between the light and dark zones. The bright zone was illuminated with white light, LED luminaire (14 watts). The dark area presents the entire structure in black acrylic and without lighting. All three cohort of mice were placed individually in the light zone of the LDT box to explore the apparatus for five minutes. Mice that display anxiety-like behavior spend less time in the light zone and perform fewer transitions compared to non-anxious control mice. The period in the light zone and the number of transitions were manually registered by a blinded treatment researcher observing video recordings of sessions. An outlier from the INF group (145 dpt, cohort 2) was excluded based on the Grubb’s test.

### Statistical analysis

Results are expressed as measures of mean ± standard errors or deviations (parasitemia levels) of the mean. The D’Agostino Pearson test was used to verify the Gaussian distribution. One-way analysis of variance (ANOVA) followed by Bonferroni’s multiple comparison *post hoc* test was used for comparisons between more than two groups, while two-way analysis of variance followed by Bonferroni’s multiple comparison *post hoc* test was used to compare the total distance traveled between groups and between sessions (repeated measures) in OFT and exploration time (repeated measures) in NORT. For comparisons between two groups, Student’s t-tests with Welch’s correction (when applicable) were used. Outliers were removed based on Grubb’s test. Differences with p values ​​less than 0.05 were considered statistically significant. GraphPad Prism 8.01 (La Jolla, CA, USA) was used to prepare charts and statically analysis.

## Results

We have previously studied behavioral aspects at 77 days after the end of CQ treatment (dpt). Thus, in this study, to further characterize the evolution of cognitive performance after a single episode of nSM in the mice, we evaluated performance of mice in the OFT and NORT at 12 dpt (cohort 1) and 145 dpt (cohorts 2 and 3).

### Non-severe *Plasmodium berghei* ANKA infection in mice impairs mice behavioral performance at 12 days after the end of treatment

All mice infected with 10^6^ pRBC by *Pb*A showed low levels of parasitemia (mean of 2.8%) four days after infection (**Figure S1**). Parasitemia was consistently negative at D10 after CQ treatment and after behavioral assays (100% of mice). CQ treatment did not affect short-term (12 dpt) performance in any behavioral paradigms OFT, NORT and LDT tested here (**Figure S2**).

**Figure 3:**
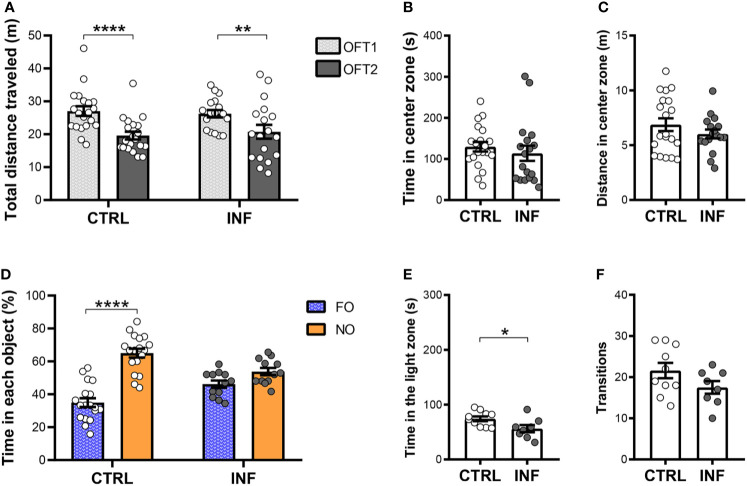
Effect of non-severe *Plasmodium berghei* ANKA infection in mice in behavioral performance in different paradigms 12 days after the end of treatment (dpt). **(A)** Total distance traveled in meters (m) in open field test (OFT1 and OFT2). **(B)** Time in center zone (seconds, s). **(C)** Distance in center zone (m) in first session of OFT. **(D)** Time in objects in percentage (%) in test session of the NORT. **(E)** Time in the light zone (s). **(F)** Numbers of transitions between light and dark side in light/dark task. CTRL (control) and INF (infected and treated) mice groups. Data points are identified as individuals’ values. Columns represent mean ± S.E.M. Two-way RM ANOVA/Bonferroni was used for intragroup comparison of different sessions (n=18-20/group) and different objects (n=12-18/group). Two-way ANOVA was used for comparison between CTRL vs INF of OFT1 and OFT2, and the p-values were non-significant (n=18-20/group). Unpaired student t-test was used to compare control and infected group (n= 8-20/group). *p<0.05; **p<0.01; ****p<0.0001.

Non-severe *Pb*A-infection had no effect on long-term habituation memory (**Figure 3A**). *Pb*A-infected and treated mice (herein referred to as infected group, INF) showed no difference neither in the total distance traveled (locomotion) in the first OFT session (OFT1), nor in OFT2, as compared to their respective control 12 dpt (**Figure 3A**). Additionally, analyzing mouse habituation to novelty performance by comparing the OFT1 and OFT2 distance traveled, mice from both CTRL and INF groups showed decreases in the OFT2 distance traveled when compared with the respective OFT1 (**Figure 3A**). To characterize the dynamics of anxiety-like behavior following an episode of nSM, cohort 1 was evaluated in the OFT1 and LDT. During the OFT1, no difference between the CTRL and INF groups was detected in the time spent in the center zone (**Figure 3B**). In the same way, no alteration was detected in distance traveled in the center zone between CTRL and INF groups (**Figure 3C**).

Non-severe *Pb*A-infection in mice induces long-term recognition memory impairment, detectable by the NORT, even after prompt radical cure by CQ. In the training session, no significant difference in the percentage of time spent exploring each object was observed between familiar objects (**Figure S3A**). Twenty-four hours after the NORT training session, no difference in object (FO and NO) exploration was observed in INF mice. INF mice were unable to distinguish the familiar object (FO) from the novel object (NO). In the test session, as expected, CTRL mice spent more time exploring the NO than FO (**Figure 3D**). The CTRL group at the 12 dpt cohort presented an average time of objects exploration of 35 seconds (s) in each session and the INF group presented 39 s.

Non-severe *Pb*A-infection in mice induces anxiety-like behavior. In the LDT, the INF group spent less time in the light zone when compared to the respective CTRL (**Figure 3E**). No difference in the number of transitions between zones was observed between INF and CTRL groups (**Figure 3F**).

### Non-severe *Plasmodium berghei* ANKA infection in mice impairs behavioral performance at 145 dpt

**Figure 4:**
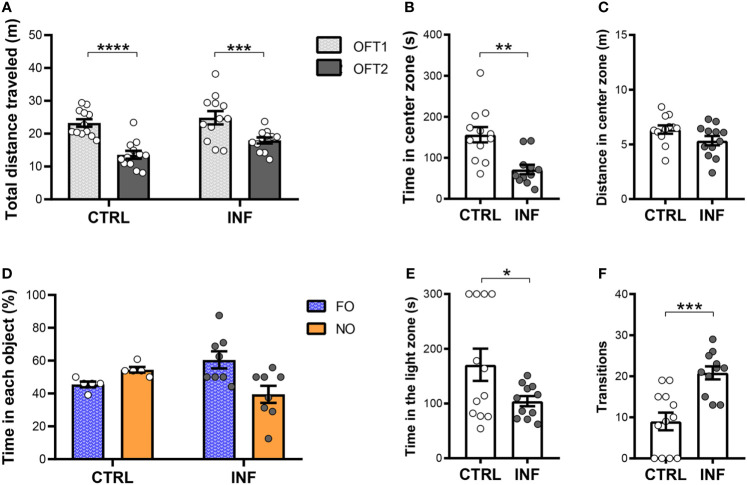
Effect of non-severe *Plasmodium berghei* ANKA infection in mice in behavioral performance in different paradigms 145 days after the end of treatment (dpt). **(A)** Total distance traveled in meters (m) in open field test (OFT1 and OFT2) 50 × 50 × 50 cm arena. **(B)** Time in center zone (seconds, s). **(C)** Distance in center zone (m) in first session of OFT. **(D)** Time in objects in percentage (%) in test session in the NORT. **(E)** Time in the light zone (s). **(F)** Numbers of transitions between light and dark side in light/dark task. CTRL (control) and INF (infected and treated) mice groups. Data points are identified as individuals’ values. Columns represent mean ± S.E.M. Two-way RM ANOVA/Bonferroni was used for intragroup comparison of different sessions (n=12/group) and different objects (n=5-8/group). Two-way ANOVA was used for comparison between CTRL vs INF of OFT1 and OFT2, and the p-values were non-significant (n=12/group). Unpaired student t-test and Welch’s correction when applicable were used to compare control and infected group (n=11-12/group). *p<0.05; **p<0.01; ***p<0.001; ****p<0.0001.

To further characterize the evolution of cognitive performance after a single episode of nSM, mice were also evaluated 145 days after the end of CQ treatment (cohort 2) in the OFT and NORT (50 × 50 × 50 cm OF arena). The INF mice showed no difference neither in the total distance traveled (locomotion) in the first OFT session (OFT1), nor in OFT2, as compared to their respective control (**Figure 4A**). Additionally, mice from CTRL and INF groups showed decreases in the distance traveled in OFT2 when compared to OFT1 and, thus, similar performance in habituation to novelty (**Figure 4A**). Non-severe *Pb*A-infection in mice induces anxiety-like behavior. Mice were evaluated in the OFT1 and LDT. INF mice spent a shorter time in the center zone, as compared to CTRL (**Figure 4B**). No alteration was detected in terms of distance traveled in the center zone between CTRL and INF mice (**Figure 4C**).

In the NORT training session, no significant difference in percentage of time spent in the exploration of each object was observed between familiar objects (**Figure S3B**). In the test session, CTRL mice spent more time exploring the NO, but no significant exploration difference was detected between NO and FO (**Figure 4D**). No difference in object exploration was observed in INF mice. CTRL mice from cohort 2 (at 145 dpt) showed a lower mean exploration time of the objects (9 s) than those from cohort 1 (at 12 dpt) CTRL group. In addition, there was an apparent reduction in locomotor activity with aging of the mice in both NORT sessions in the CTRL groups [28 and 13 meters average total distance traveled in the training session and of 21 and 10 meters in the test session, at 12 and 145 dpt (cohort 2) respectively, **Figure S4**].

In the LDT, the INF mice spent less time in the light zone when compared to CTRL (**Figure 4E**). Mice from the CTRL group showed a fewer number of transitions as compared to INF mice (**Figure 4F**).

**Figure 5:**
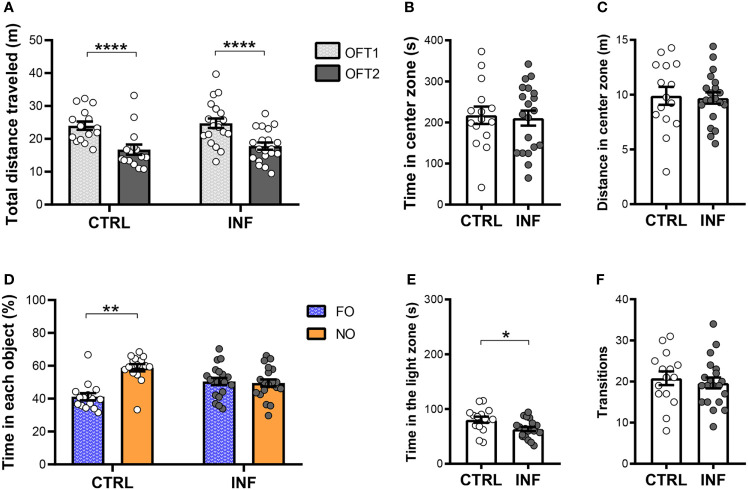
Effect of non-severe *Plasmodium berghei* ANKA infection in mice in behavioral performance in different paradigms 145 days after the end of treatment (dpt). **(A)** Total distance traveled in meters (m) in open field test (OFT1 and OFT2) 30 × 30 × 30 cm arena. **(B)** Time in center zone (seconds, s). **(C)** Distance in center zone (m) in first session of OFT. **(D)** Time in objects in percentage (%) in test session in the NORT. **(E)** Time in the light zone (s). **(F)** Numbers of transitions between light and dark side in light/dark task. CTRL (control) and INF (infected and treated) mice groups. Data points are identified as individuals’ values. Columns represent mean ± S.E.M. Two-way RM ANOVA/Bonferroni was used for intragroup comparison of different sessions (n=15-20/group) and different objects (n=15-20/group). Two-way ANOVA was used for comparison between CTRL vs INF of OFT1 and OFT2, and the p-values were non-significant (n=15-20/group). Unpaired student t-test was used to compare control and infected group (n=15-20/group). *p<0.05; **p<0.01; ****p<0.0001.

Due to the low average exploration of cohort 2 objects in the NORT when compared to cohort 1, analyzes with mice from cohort 3 (145 dpt) were performed in a smaller apparatus in OFT and NORT (30 × 30 × 30 cm OF arena). The INF mice showed no difference neither in the total distance traveled (locomotion) in the first OFT session (OFT1), nor in OFT2, as compared to their respective controls (**Figure 5A**). Additionally, mice from both groups CTRL and INF showed a decrease in distance traveled in OFT2 when comparing to OFT1 (**Figure 5A**). Mice from cohort 3 were evaluated at 145 dpt in the OFT1 and LDT for the anxiety-like behavior. During the OFT1, CTRL and INF mice spent similar time in the center zone of the apparatus (**Figure 5B**). In the same way, no alteration was detected in distance traveled in the center zone between CTRL and INF groups (**Figure 5C**).

In the NORT training session, no significant difference in percentage of time spent in the exploration of each object was observed between familiar objects (**Figure S3C**). In the test session, CTRL mice spent more time exploring the NO, showing exploration significant preference for NO over FO (**Figure 5D**). No differences in object exploration were observed in INF mice. INF mice were unable to distinguish the NO from the FO. The 145 dpt cohort 3 CTRL group showed a mean exploration time of 42 seconds (s) in each session as compared to 9 s of the cohort 2 CTRL group.

Non-severe *Pb*A-infection in mice induces anxiety-like behavior. In the LDT, the INF mice spent less time in the light zone compared to CTRL mice (**Figure 5E**), although no difference was observed in the number of zone transitions between INF and CTRL mice (**Figure 5F**).

### Immunization with dT vaccine reverses behavioral impairment induced by non-severe *Plasmodium berghei* ANKA infection in mice

**Figure 6:**
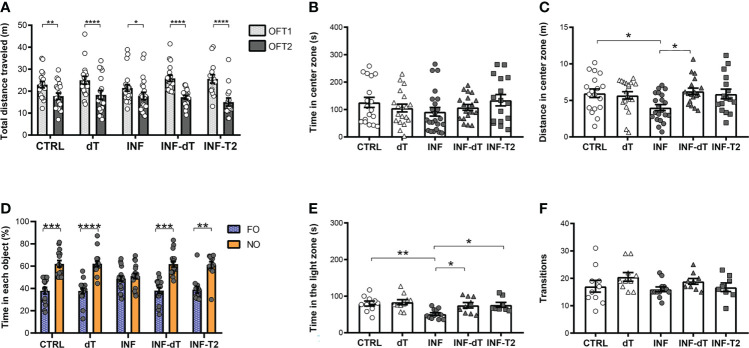
Immunization effect on behavioral performance in different paradigms of health mice and after non-severe experimental malaria (control (CTRL), immunized control with dT vaccine (dT), infected (INF), immunized infected with dT vaccine (INF-dT) and immunized infected with T2 stimuli (INF-T2) mice). **(A)** Total distance traveled in meters (m) in open field test (OFT1 and OFT2). **(B)** Time in center zone (seconds, s). **(C)** Distance in center zone (m) in first session of OFT. **(D)** Time in objects in percentage (%) in test session in the NORT. **(E)** Time in the light zone (s). **(F)** Numbers of transitions between light and dark side in light/dark task. Data points are identified as individuals’ values. Columns represent mean ± S.E.M. Two-way RM ANOVA/Bonferroni was used for intragroup comparison of different sessions (n=15-22/group) and different objects (n=13-19/group). Two-way ANOVA was used for intergroup comparison of the same sessions (n=15-22/group). One-way ANOVA/Bonferroni was used for comparison of CTRL/INF, CTRL/INF-dT, CTRL/INF-T2, INF/INF-dT e INF/INF-T2 (n= 8-23/group). The p-values between CTRL and INF-dT and INF-T2 were non-significant. *p<0.05; **p<0.01; ****p<0.0001.

We investigated whether immunization with the dT vaccine alone had a positive impact on behavioral parameters and, if so, whether this effect was comparable to that obtained with integrated immune stimuli capable of inducing T2 responses, as previously reported (18). dT vaccine immunization does not affect habituation memory or locomotor activity. In the first OFT session (OFT1), the total distance traveled by dT, INF, INF-dT and INF-T2 groups of mice, chosen for the locomotor activity analysis, was not different from the CTRL. The CTRL, dT, INF, INF-dT and INF-T2 groups of mice traveled a significantly shorter mean distance in the OFT2 session compared to the OFT1 (**Figure 6A**).

The effect of immunization on anxiety-like behavior was also assessed in CTRL, dT, INF, INF-dT and INF-T2 groups of mice by OFT and LDT. The time spent in the center zone of the apparatus by dT, INF, INF-dT and INF-T2 groups was not different from that of the CTRL in OFT1 (**Figure 6B**). INF mice traveled a shorter distance in the center of the apparatus as compared to CTRL (**Figure 6C**). The immunized groups (dT, INF-dT and INF-T2), in addition to not showing differences in this parameter in relation to the CTRL, did not differ from each other. However, INF-dT mice traveled a greater distance in the center zone as compared to INF (**Figure 6C**).

### dT vaccination reverses long-term recognition memory impairment induced by non-severe *Plasmodium berghei* ANKA infection in mice

To assess whether the dT vaccine immunization can recover the long-term memory deficit detected after nSM, the exploration time between objects in percentage was estimated in the NORT at 77 dpt. In the training session, different groups of mice explored the familiar objects FO1 and FO2 in a similar way and no significant differences in exploration were observed between objects (**Figure S5**). After 24 hours, in the test session, dT, INF-dT and INF-T2 mice showed a significantly greater exploration of NO than of FO, as observed for the CTRL (**Figure 6D**). Mice from INF group showed no preference for the NO. They were unable to distinguish the NO from the FO and, explored both for approximately the same time.

### The dT vaccine-immunization reverses anxiety-like behavior induced by non-severe *Plasmodium berghei* ANKA infection in mice

In the LDT, the time spent in the light zone and the number of transitions were counted in the same experimental groups mentioned above. INF mice spent a shorter time in the light zone of the apparatus in comparison to CTRL (**Figure 6E**). The immunized groups (dT, INF-dT and INF-T2), in addition to not showing differences in this parameter in relation to the CTRL, did not differ from each other. However, INF-dT and INF-T2 mice spent a greater time in the light zone as compared to INF. No significant differences were detected in the number of transitions between the groups evaluated (**Figure 6F**).

## Discussion

Non-severe malaria (nSM) caused by *Plasmodium falciparum* is the most common presentation of malaria. In recent decades, evidence has accumulated showing the impact of nSM on children’s cognitive function, mainly in the reduction of school performance (5, 8, 27–29). The classical experimental models of nSM (as, for example, in Swiss Webster mice infected with non-lethal *P. yoelii*, C57BL/6 with *P. chabaudi chabaudi* or *P. chabaudi adami*) have not reproduced the cognitive deficits recorded in man (3, 30). However, behavioral changes have been reported as sequelae of nSM in experimental models only recently (6, 18, 31). De Sousa et al. studied the classical experimental model of CM (C57BL/6 infected with *P. berghei* ANKA) treated with CQ before any clinical signs of CM and observed late behavioral changes and cognitive deficits (6, 18).

The cognitive deficits of nSM may be persistent, but the evolution of cognitive and behavioral impairment is poorly understood (12, 28). Persistent cognitive impairment may affect social, economic and cultural development in malaria-endemic areas. In this work, experiments in the murine model were carried out to evaluate the cognitive and behavioral performance 12 (short-term) and 145 dpt [to assess persistence using a longer period than the long-term (77 dpt), evaluated by De Sousa et al. (6, 18)].

In the OFT, no impact on locomotion activity in mice after nSM was detected at any of the different times evaluated in all three cohorts, in agreement with the observations made after treatment (6, 18) and even during active infection (30) of non-severe experimental malaria (nSEM).

The OFT is a widely used test to assess locomotor activity, habituation memory, and anxiety-related parameters (22, 32). Long-term memory deficits related to novelty habituation were not detected by the OFT in any of the cohorts analyzed after CQ treatment. These data corroborate those by De Sousa et al. (6, 18) showing that this kind of deficit was absent 77 days after parasite clearance.

Object recognition deficits can also result from nSEM and were observed here as early as 12 dpt and can persist for up to nearly five months (145 dpt) after treatment. The cohort of older mice tested in 50 × 50 × 50 cm apparatus (145 dpt) showed less exploration in the NORT compromising the analysis. The use of a 30 × 30 × 30 cm apparatus for the behavioral tasks allowed greater exploration of the objects by the mice, enabling more reliable analysis. Aging modulates many aspects of behavior in a continuous manner in C57BL/6 mice (33). Therefore, differences in the distance traveled in OFT and transitions in LDT in older mice could explain the changes in the exploratory behavior of cohort 2 compared to cohort 1. Long-term recognition memory in rodents, an important tool that models humans’ ability to recognize objects, individuals and events, was assessed by the NORT task (34). Deficits in long-term recognition memory were detected during experimental cerebral malaria (ECM) by De Miranda et al. (35) and Campos et al. (36), three days after the end of ECM treatment with CQ (3), and seven days after treatment with artesunate (36). One brain structure associated with recognition memory deficits in humans and rodents is the hippocampus (37–39). Their detection after nSM is of relevance, since it shows that alterations in this task are not restricted to CM. No deficits in long-term recognition memory was detected at the peak of the parasitemia curve during infection of C57BL/6 mice infected with *P. chabaudi adami*, a classical experimental model of nSM (30). However, late term memory deficits (77 dpt with CQ) were detected in nSEM (6, 18). Learning and memory are necessary for the acquisition, retention and reconstruction of knowledge about the world over time (40).

Anxiety-related parameters were evaluated after nSEM treatment in two different tests (OFT and LDT) in INF groups from all cohorts. Among the predictive behavioral tests to explore anxiety-like behavior, the LDT is considered a robust test to assess this complex phenotype in rodent (41), and the time spent in the light compartment is the main predictive parameter used in most studies in the literature, instead of the number of transitions (42). In this sense, increases in time spent in the light zone by INF groups have been observed here in all three cohorts in LDT. Contrasting with other cohorts, CTRL mice from cohort 2, evaluated at 145 dpt, showed a lower number of transitions between the light and dark compartments compared to INF mice. As mentioned before, ageing could affect mice’s behavioral performance (33) and the stress stimuli induced by the behavioral protocol used in cohort 2 (a larger OFT arena – 50 × 50 × 50 cm) could exemplify this confounding effect observed between the light and dark compartment transitions. Additionally, behavioral alterations have also been described recently in mice with history of juvenile malaria, in response to stress stimulus at adulthood (31). However, our group was the first to show behavior sequelae in nSEM (6, 18) as well as their presence in the short-term (12 days) post-treatment, and their persistence for up to 145 days, in this work.

Malaria is a systemic infection, the pathology of which may be influenced by several aspects of the parasite-host relationship, including parasite and host genetic background, the nature and intensity of the infection, and induced immune response (6, 19, 20, 43, 44). From an immunological point of view, during the erythrocyte cycle of *Plasmodium* development, parasite and host cell antigens are released into the bloodstream with the rupture of erythrocytes, activating an immune response. An increase in pro-inflammatory mediators, such as IFN-ɣ and TNF-α, contributes to the immune response, microglial activation, and polarization of the immune response towards the Th1 profile (20, 45–48) and may contribute to acute and late cognitive deficits of CM in children (49).

Pathological outcomes manifesting during the early stages of infection can lead to changes in the CNS microenvironment and may culminate in the deregulation of homeostasis of cognitive functions. Therefore, even the minimal cerebral changes that may occur during asymptomatic (according to Khandare et al.) phases of infection may contribute to the results reported here (50). For example, on the third day post infection, increased expression of neuronal activation marker c-fos protein (51), increased cerebellar gene expression of programmed cell death proteins 1 (PD-1), lymphocyte activation gene 3 (LAG-3) and T lymphocyte-associated protein 4 (CTLA-4), and reduction of hippocampal chemokine ligand 4 (CXCL-4) occur (50). On the fourth day post-infection increased levels of TNF cytokine in brain cortical tissue (52), adhesion of leukocytes and the minimal presence of edema restricted to a focal area in the brain have been observed (21).

Both infection and immunization are capable of altering immune and cognitive responses. De Sousa et al. (18) showed that type 2 (Th2) immune stimuli can positively modulate the neurocognitive response in malaria and reverse the cognitive deficits associated with nSM. Here, we investigated the effect of isolated immunization with the dT vaccine by comparing it to type 2 integrated stimuli effect, demonstrated by De Sousa et al. at 77 dpt (18).

The dT vaccine corresponds to one of the integrated type 2 stimuli and was chosen because: a) it is a known inducer of Th2 immune response (53–55); b) it contains the aluminum phosphate (AlPO_4_) adjuvant which also has a type 2 immunomodulatory effect (56); and c) it is included in the list of vaccines offered by the *Programa Nacional de Imunizações* (*Ministério da Saúde*, BR) contributing to a translational applicability and has been applied worldwide in billions of doses in recent decades.

Immunization with the dT vaccine, with a 21-day interval, started 14 days after the end of CQ treatment of non-severe *Pb*A malaria episodes, promoted the reversal of memory deficits of long-term recognition in NORT and anxiety-like behavior in OFT and LDT. As there was no difference in the time spent in the center zone between all groups analyzed, as De Sousa et al. (18) also noted, anxiety parameters need to be further investigated through other tests, such as the Elevated Plus Maze. However, the results obtained in this work indicate that the dT vaccine, administered alone after CQ treatment, may have an anxiolytic-like effect in mice with nSM.

Such effects, as well as those obtained with type 2 integrated stimuli previously reported by De Sousa et al. (18), also concerning memory and anxiety alterations recorded after a single episode of nSM, indicate that immunization, done after malaria episodes, is capable of reversing (but not preventing) cognitive and behavioral deficits, since these sequelae are already evident early (12 days) after treatment. We have observed that the experimental model of nSM immunized with T2 stimuli showed an increase in regulatory T cells in the spleen and IL-10 in the serum that could act by controlling neuroinflammation and contributing to the effects that promote cognitive function (18). Behavioral effects resulting from immunization with the dT vaccine alone may operate through the same mechanism. The present study did not analyze cytokine levels and T cell polarization to confirm Th2 cytokine production due to dT vaccination or relate these parameters to behavioral indices measured. Studies investigating immune cells and mediators, including the profile of cytokines secreted by regulatory T cells in serum and hippocampus at different times post infection, are necessary to increase the understanding of mechanisms behind the deficit recovery effect of immune stimuli and may help the development of strategies for the recovery of cognitive ability in children and residents of malaria-endemic areas, and even in other conditions that affect cognition.

## Conclusion

Poor performance on behavioral tests is detectable in mice with nSM as early as 12 days after CQ treatment and may persist for five months. These results seem to justify the implementation of strategies to recover cognitive and behavioral deficits resulting from nSM soon after the end of antimalarial treatment in children or elderly patients. Immunization with the dT vaccine seems to have effects comparable to those observed with T2 stimuli and may constitute a promising strategy for reversing cognitive and behavioral alterations associated with human malaria. Clinical assays could help in defining the viability of these measures.

## Data availability statement

The original contributions presented in the study are included in the article/**Supplementary Material**. Further inquiries can be directed to the corresponding author.

## Ethics statement

The animal study was reviewed and approved by ethics committee of the Instituto Oswaldo Cruz (CEUA-IOC, Fiocruz, license numbers L-010/2015, L-004/2020).

## Author contributions

PR-G conducted the experiments, analyzed, interpreted the data and wrote the manuscript. AM assisted in performing the experiments. LS started the immunomodulation research line in nSM and taught the technique of behavior and immunization protocols in the initial experiments. FR-G helped with the protocols, followed the experiments and contributed to the discussion. CG assisted in performing the experiments. GW chose the statistical tests that were performed, and supervised the work done by PR-G. DS helped with data analysis and interpretation. RA helped with experimental design, behavioral protocols, data analysis, interpretation and discussion. CD-R conceived the study, interpreted the data, reviewed and edited the manuscript versions. All authors contributed to the article and approved the submitted version.

## Funding

PR-G, AM, CG were supported by the *Conselho Nacional de Desenvolvimento Científico e Tecnológico (CNPq)* and *Instituto Oswaldo Cruz (IOC), Fiocruz*, students’ fellowships. FR-G, GW, DS and CD-R are supported by the *CNPq*, Brazil, through a Productivity Research Fellowship, and GW and CD-R are *Cientistas do Nosso Estado* by the *Fundação Carlos Chagas Filho de Amparo à Pesquisa do Estado do Rio de Janeiro (Faperj)*. The *Laboratório de Pesquisa em Malária*, *(IOC)*, *Fiocruz* is an Associated Laboratory of the *Instituto Nacional de Ciência e Tecnologia (INCT) em Neuroimunomodulação* supported by the *CNPq* (Project *INCT-NIM* 465489/2014-1), and of the *Rede de Neuroinflamação do Rio de Janeiro* financed by *Faperj* (Project *Redes/Faperj* 26010.002418/2019). This work was partially supported by the Project SEI-260003/001169/2020 of the *Faperj Programa de Apoio a Projetos Temáticos no Estado do RJ* 2019 to CTD-R and by the *POM* of *IOC, Fiocruz*.

## Acknowledgments

PR-G is grateful to the Graduate Program in Parasite Biology of the *Instituto Oswaldo Cruz (IOC)*, *Fiocruz*. The work described in this manuscript extends parts of the MSc dissertation of PR-G (2021) entitled: Dinâmica e imunomodulação de déficits cognitivos e alterações comportamentais da malária não grave experimental. [Rio de Janeiro (RJ)]: Fiocruz. The authors are grateful to Dr. Richard Culleton for critical English-language review and for the valuable suggestions. The authors thank Dr. Mônica Nogueira and her team of the *Centro de Experimentação Animal* (IOC, Fiocruz) for caring for the animals and supporting the experiments. We thank Dr. Tadeu Mello e Souza (from UFRGS) for the rich discussions on the behavioral and cognitive tests. We thank the Multi-user Research Facility of Flow Cytometry, IOC, Fiocruz, Rio de Janeiro, Brazil. The authors are thankful to Dr. Aline Moreira, MSc Fabiana Gomes, Guilherme Salgado, Bruno Vasconcelos (from IOC, Fiocruz) for training PR-G on the experimental model. We are grateful to Drs. Rudimar Frozza (from IOC, Fiocruz); Andréa Lucas and Juliana Medeiros (from Farmanguinhos, Fiocruz); Dr. Maria de Lourdes de Sousa Maia (from Bio-Manguinhos, Fiocruz); Dr. Pierre Druilhe (from Vac4All Initiative – Paris/France) for sharing reagents and equipment. *Plasmodium berghei*, Strain (ANKA) GFPCON 259cl2, MRA-865, was obtained through BEI Resources, NIAID, NIH and was contributed by Chris J. Janse and Andrew P. Waters.

## Conflict of interest

The authors declare that the research was conducted in the absence of any commercial or financial relationships that could be construed as a potential conflict of interest.

## Publisher’s note

All claims expressed in this article are solely those of the authors and do not necessarily represent those of their affiliated organizations, or those of the publisher, the editors and the reviewers. Any product that may be evaluated in this article, or claim that may be made by its manufacturer, is not guaranteed or endorsed by the publisher.
