# Supplementary material for: Dynamics and immunomodulation of cognitive deficits and behavioral changes in non-severe experimental malaria
Source: Front Immunol. 2022 Nov 24;13:1021211. doi: 10.3389/fimmu.2022.1021211 (PMC9729266; doi:10.3389/fimmu.2022.1021211)
Supplement: Supplementary file 1 [file DataSheet_1.pdf]

Supplementary Material

1 Supplementary Figures

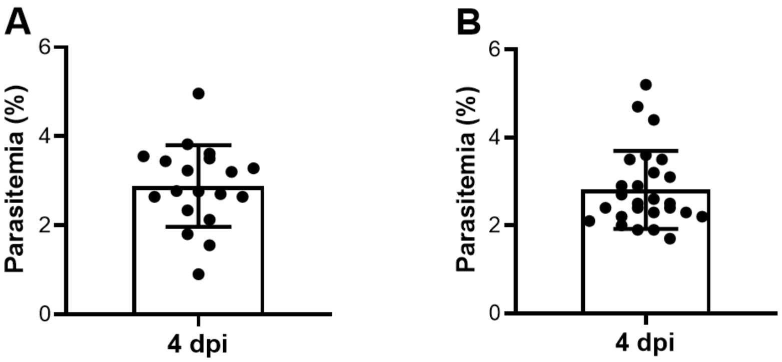

Figure S1. Parasitemia levels in percentage values four days after infection with *Plasmodium berghei* ANKA. **(A)** Percentage of parasitized red blood cells measured by slide distention (n=19). **(B)** Percentage of parasitized red blood cells GFP+ measured by flow cytometry (n=26). Data points are identified as individual's values. Columns represent mean ± S.D.

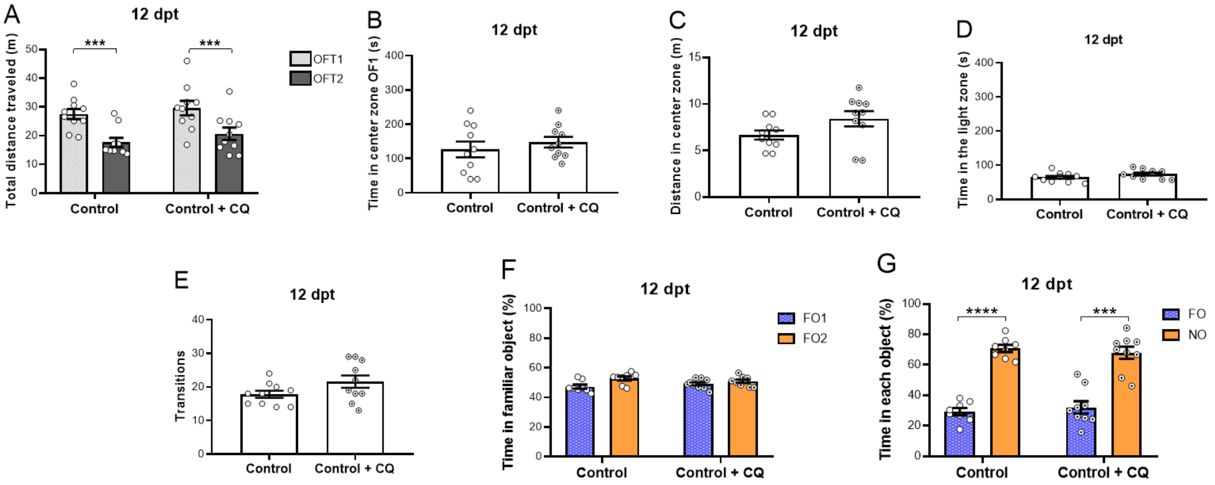

Figure S2. Effect of chloroquine treatment on different paradigms of naive mice 12 days after the end of treatment (dpt). **(A)** Total distance travelled in meters (m) in the open field test (OFT1 and OFT2). **(B)** Time in center zone (seconds, s). **(C)** Distance in center zone (meters, m) in the first session of OFT. **(D)** Time in the light zone (seconds, s). **(E)** Numbers of transitions between light and dark side in light/dark task (LDT). **(F)** Time in familiar objects in percentage (%) in training session of novel object recognition task (NORT). **(G)** Time in objects in percentage (%) in test session. Data points are identified as individual's values. Columns represent mean ± S.E.M. Two-way RM ANOVA/Bonferroni was used for intragroup comparison of different sessions and different objects. Two-way ANOVA was used for comparison between Control vs Control + CQ of OFT1 and OFT2, and the p-values were non-significant. Unpaired t-Student test was used to compare two groups (n= 10/group). (***) p<0.001;

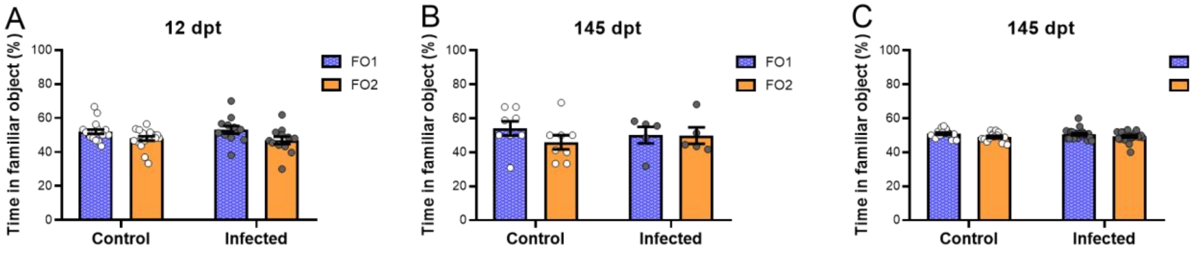

Figure S3. Evolution of long-term recognition memory of mice after non-severe experimental malaria in training session of novel object recognition task (NORT). Time in familiar objects in percentage (%) of infected and control mice in training session, lasting 10 minutes in the NORT. **(A)** Performance of cohort 1 evaluated 12 days post-treatment (dpt). **(B)** Performance of cohort 2 evaluated 145 dpt. **(C)** Performance of cohort 3 evaluated 145 dpt. Values were manually timed with digital stopwatch. Data points are identified as individual's values. Columns represent mean ± S.E.M. Two-way RM ANOVA/Bonferroni was used for intragroup comparison of different objects (n= 5-20/ group). (*) p<0.05; (***) p<0.001.

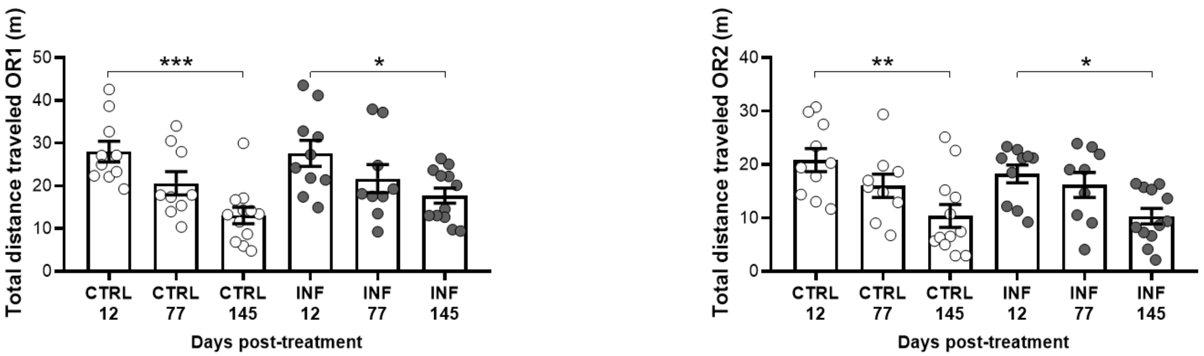

Figure S4. Total distance travelled in the novel object recognition task (NORT) 12 (cohort 1), 77 and 145 dpt (cohort 2) of mice cohorts after non-severe experimental malaria. **(A)** Training session. **(B)** Test session. One-way ANOVA/Bonferroni was used for comparison of CTRL 12 dpt/ CTRL

77 dpt/ CTRL 145 dpt and INF 12 dpt/ INF 77 dpt/ INF 145 dpt (n=9-12/group). (*) p<0.05; (**) p<0.01; (***) p<0.001.

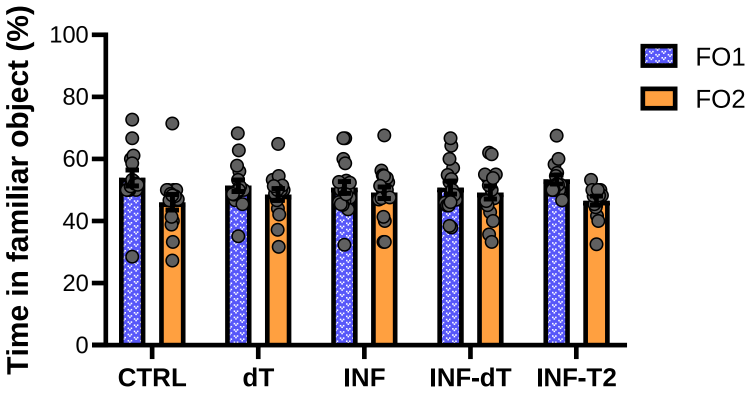

Figure S5. Immunization effect on long-term recognition memory of mice after non-severe experimental malaria in training session of novel object recognition task (NORT). Time in familiar objects in percentage (%) of control (CTRL), immunized control with dT vaccine (dT), infected (INF), immunized infected with dT vaccine (INF-dT) and immunized infected with T2 stimuli (INF-T2) mice in training session, lasting 10 minutes in the NORT. Values were manually timed with digital stopwatch. Data points are identified as individual's values. Columns represent mean ± S.E.M. Two-way RM ANOVA/Bonferroni was used for intragroup comparison of different objects (n= 13-19/ group).
